# Managing the premenstrual body: a body mapping study of women’s negotiation of premenstrual food cravings and exercise

**DOI:** 10.1186/s40337-021-00478-6

**Published:** 2021-10-09

**Authors:** Samantha Ryan, Jane M. Ussher, Alexandra Hawkey

**Affiliations:** Translational Health Research Institute, Western Sydney University, Locked Bag 1797, Penrith South DC, NSW 2751 Australia

**Keywords:** Premenstrual distress, Body dissatisfaction, Body management, Disordered eating, Exercise, PMS, Restrictive eating, Premenstrual

## Abstract

**Background:**

Women’s eating behaviours and exercise patterns have been found to fluctuate across the menstrual cycle, manifested by premenstrual food cravings and reduced exercise. However, the meaning and consequences of premenstrual changes in eating and exercise behaviours remains underexplored. The aim of this qualitative study was to explore how women who feel negatively about their premenstrual bodies construct and experience premenstrual changes to eating and exercise practices, which disrupt their usual patterns of body management.

**Methods:**

Four hundred and sixty women aged 18–45 completed an online survey in response to a Facebook advertisement targeted at women who feel negatively about their bodies during the premenstrual phase of the cycle. Participants reported moderate premenstrual distress, high body shame and high risk of disordered eating attitudes using standardised measures. Sixteen women reporting rich accounts of premenstrual body dissatisfaction were invited to participate in body-mapping, involving visually illustrating experiences on a life-sized outline of the body, followed by a telephone interview. Thematic analysis was used to explore qualitative survey, interview, and body-mapping data.

**Results and discussion:**

Results found that outside of the premenstrual phase these women engaged in restrictive eating and intensive exercise behaviours, which were disrupted by premenstrual cravings, hunger, fatigue, pain and feeling physically uncomfortable. For a minority of the women, this facilitated self-care in reducing the strict management of their bodies during the premenstrual phase. Others experienced feelings of guilt, shame, self-disgust and pushed their bodies physically through increased exercise.

**Conclusions:**

These findings emphasise the need to acknowledge changes in body management across the menstrual cycle, with implications for women’s mental health and feelings about the self. Internalisation of pressures placed on women to manage their bodies through restrictive eating behaviours and rigorous exercise plays a role in women’s premenstrual body dissatisfaction and distress.

**Plain English summary:**

The current study aimed to explore how women who feel negatively about their premenstrual bodies construct and experience premenstrual changes to eating and exercise practices. Outside of the premenstrual phase these women engaged in restrictive eating and intensive exercise behaviours which were disrupted by premenstrual cravings, hunger, fatigue, pain and feeling physically uncomfortable. Some women allowed themselves to take a premenstrual break from their usual strict eating and exercise behaviours, whereas others felt guilt, shame, self-disgust and physically pushed their bodies through increased exercise. These findings emphasise that changes to eating and exercise behaviours across the menstrual cycle and pressures placed on women to manage their eating and exercise behaviours have implications for women’s premenstrual distress and body dissatisfaction.

## Background

Within Western culture, women are continually bombarded with messages from the media that position their bodies as defective and receive instructions regarding how their bodies should look and be managed [1, 2]. As a result, pressure is placed upon women to control, manage and discipline their bodies, through constant self-surveillance and regulatory behaviours [3]. Many women strive to achieve the ideal Western feminine body—the thin, toned, attractive, controlled body—and position the fat body as unhealthy, lazy, undisciplined and out of control [4, 5]. Appetite for food is a drive women are expected to resist and control, with unrestrained eating positioned as unfeminine and inappropriate for women [6]. Instead, women are encouraged to engage in a range of dieting behaviours, such as calorie counting, food weighing and restrictive eating [7]. At the same time, women are expected to engage in regular and rigorous exercise, behaviours positioned within popular culture as healthy and necessary to prevent their bodies from becoming fat and ‘out of control’ [1, 8]. Normalisation of these behaviours within the media can be problematic for women, encouraging disordered eating, unhealthy exercise behaviours and body dissatisfaction [9–11]. Body dissatisfaction and failure to meet these ideals has negative implications for mental health, associated with increased risk of depression and low self-esteem [12–14].

Within popular culture and medical texts women are discursively positioned as out of control during the premenstrual phase of the menstrual cycle, with one facet of this discursive positioning being representations of the ‘insatiable’ appetite of premenstrual women [15, 16]. Previous research has found that women’s eating behaviours fluctuate across the menstrual cycle, associated with reports of elevated food intake and increased premenstrual cravings for food [17, 18]. Women have also been found to have heightened reactivity to food cues during the premenstrual phase, suggesting that it may become more difficult to practice restrained eating during this time [19]. Specifically, consumption of carbohydrates have been found to be highest during the premenstrual phase [18], as have cravings for sweet and fatty foods [20]. A physiological component is suggested to be associated with premenstrual food cravings, including an increased metabolism when progesterone and estrogen are at its peak [18, 21–23]. Decreased serotonin levels during the premenstrual phase may also increase cravings for serotonin releasing foods [24]. An alternative explanation is that women are craving forbidden foods positioned as unhealthy and thus restricted within a ‘healthy’ feminine diet [25] and being premenstrual may be represented as an acceptable time for women to break these food-related restrictions. Women who experience cravings and increased food intake during the premenstrual phase have been found to have higher fear of fatness, food-related guilt and increased maladaptive weight and eating-related behaviour [26]. Reduced exercise during the premenstrual phase has also been reported, associated with guilt and negative feelings about the body [5].

There is evidence that women’s body dissatisfaction is highest during the premenstrual phase in the general female population [21, 27, 28]. Distorted perceptions of body size is one possible explanation, with some findings suggesting women perceive their bodies as larger premenstrually [28, 29]. In women who report premenstrual distress, levels of premenstrual symptom severity have been associated with body image disturbance [30] body shame and body dissatisfaction [5, 31, 32]. Premenstrual distress involves affective, behaviour and physical changes, such as anxiety, depression, feelings of loss of control, abdominal bloating, breast tenderness and fluid retention, described as premenstrual syndrome (PMS) [33]. Women who experience PMS commonly describe their premenstrual bodies as “fat”, “ugly”, “sluggish” and “unattractive”, value laden terms associated with reduced self-worth, self-repulsion and self-criticism [5, 34]. Given these negative self-perceptions there is a need to explore the implications of fluctuations in eating and exercise behaviours on women’s premenstrual body dissatisfaction and distress. This qualitative study examines the ways in which women construct and experience their premenstrual bodies in relation to cyclical changes in eating and exercise.

## Method

### Participants

Four-hundred and sixty cisgender women who self-identified as having an increase in negative feelings about their bodies during the premenstrual phase of the menstrual cycle took part in an online survey. Participants were aged 18–45 with a mean age of 24.1 years (*SD* = 5.85); 76.5% identified as heterosexual, 17.6% as bisexual, 2.6% as lesbian and 3.2% identified as ‘other’. Most participants were partnered (63.3%) and Anglo-Australian (Caucasian) (70.7%). Sixteen women participated in a body-mapping session and follow-up telephone interview. A previous body-mapping systematic review suggests that sample sizes of 6–12 participants are sufficient in capturing rich data relevant to research questions and a range of experiences [35].

### Materials and procedure

#### Survey

An online survey titled, ‘Premenstrual Change and Feelings Towards Your Body’, was advertised on social media, directed at women who experienced premenstrual body dissatisfaction. The Premenstrual Symptom Screening Tool (PSST) [36] is a self-report measure used to identify women who suffer from premenstrual disorders including Premenstrual Dysphoric Disorder (PMDD) and PMS, and was included as a measure of premenstrual distress. Seven of eight items on the Eating Attitudes Test 8 (EAT-8) [37] were included as a measure of disordered eating attitudes, excluding the item “I am preoccupied with the desire to be thinner” due to similarity with another question within the survey. The Objectified Body Consciousness Body Shame Subscale (OBCBSS), a subscale of the Objectified Body Consciousness Scale [38] was used as a self-report measure of body shame. Analysis of five open-ended survey questions is reported in this article. These questions asked the women if they felt differently about their premenstrual body, if their eating and exercise habits changed during the premenstrual phase, how they cope premenstrually and if they had anything else they wanted to share about their premenstrual experiences. Upon finishing the survey, participants were invited to opt-in for a body-mapping session and follow-up interview. The first author contacted participants who reported rich accounts of increased body dissatisfaction during the premenstrual phase within open-ended survey items.

#### Body-mapping

Participants in this study completed a face-to-face individual body-mapping session. Body mapping is an arts-based method involving tracing around a person’s body, creating a life-sized outline to which participants use arts supplies to fill the map in a way that artistically express an experience [39]. This method draws attention to embodied experience, encouraging bodily awareness, reflection and providing the opportunity to pinpoint areas of the body in which feelings and sensations are experienced [40].

The women were first asked to brainstorm colours, textures, words and symbols that captured experiences of their premenstrual and non-premenstrual bodies. Prompts included premenstrual changes, feelings about the body, coping and the location of these experiences, sensations or emotions within the body. The women were offered the option of having their body traced onto a large sheet of paper or to use a pre-drawn outline, an approach used in previous body-mapping research [41]. Arts supplies including paint, markers, glitter, crayons, pencils and magazines were used to visually represent experiences on the body-map. Upon completion, participants were asked to verbally describe the artistic choices they had made and why, following established protocols for body-mapping research [42]. Body maps took between 60 and 90 min and descriptions of body maps lasted between 4 and 11 min, and were audio-recorded.

#### Interviews

Semi-structured telephone interviews were conducted within five days of completing the body maps to further explore these women’s feelings about their premenstrual bodies and elaborate on descriptions of the body maps. Interviews lasted between 40 and 70 min and were audio recorded and professionally transcribed. Transcripts were integrity checked for accuracy, and all identifying information excluded. All participants were assigned a pseudonym.

### Reflexivity

Reflexivity is a process of critical self-reflection into the ways in which researchers’ social backgrounds, assumptions, positioning and behaviour may shape the research process [43]. Body-mapping sessions and interviews were conducted by the first author, SR. In describing their own premenstrual experiences during the body-mapping process, many participants asked if the researcher shared their embodied premenstrual experiences. We had agreed that it was appropriate to answer these questions honestly, without discussing the researcher’s own experiences in detail [44]. All participants informally reported positive experiences in completing their body map, allowing for expression of their embodiment in a creative and visual way, facilitating deeper understanding and self-awareness, without feelings of judgement.

### Analysis

Theoretical thematic analysis, involving searching for meaningful patterns within datasets [45], was utilised for open ended survey responses, interview and body-mapping data. We adopted a material-discursive-intrapsychic theoretical framework [46] and a critical-realist epistemology [47] which recognises the materiality of somatic, psychological and social experience but situates these experiences within cultural and historical discourse. An inductive approach was undertaken in which themes identified were data driven, and not fitted into a pre-existing coding frame [45]. The first step involved familiarization with the data through reading interview transcripts and open-ended survey responses. Initial codes included, *‘craving ‘bad’ food’* and *‘no motivation to exercise’*, which were grouped to form the basis of the coding frame. The coding frame was developed, tested and refined, and all of the data was then coded using NVivo software. Images of body-maps were coded with participant’s body-map descriptions and interview data, following recommendations by Dew, Smith (41). A coding summary was created, in which each coded set of data was summarised with reference to individual participant accounts. This was repeated with body-maps, grouping together commonalities in visual images, words and colours. The coded and summarised data was then re-examined and relationships and similarities across codes were mapped. Overarching themes and subthemes were then placed into a table, along with the corresponding data, which was further discussed and refined with the research team. Participant pseudonyms are used to present data from interviews, body-maps and verbal body-map descriptions and survey data is identified as a ‘survey participant’.

## Results and discussion

Participant’s reported moderate-severe premenstrual distress with a mean score of 22.06 on the PSST [36]. These scores were lower than scores reported by Ussher and Perz (31) in a clinical sample (*M* = 26.60). Scores on the EAT-8 indicated that participants reported high-risk of disordered eating attitudes, with a mean score of 3.62, found to be higher than scores obtained in a community sample (*M* = 1.91) [37]. Participants also indicated high levels of body shame, with a mean score of 3.89, found to be higher than scores obtained in a community sample by Sveinsdóttir [48] (*M* = 3.10). Differences in scores between the survey participants and body-mapping and interview participants can be seen in Table 1.

**Table 1:** Average age and scores of premenstrual distress, disordered eating attitudes and body shame for survey participants and body-mapping and interview participants

Variables	Survey participants (*M*)	Body-mapping and interview participants (*M*)
Age	24.01	25.50
Premenstrual Distress	21.91	26.31
Disordered Eating Attitudes	3.63	3.33
Body Shame	3.84	4.62

Thematic analysis identified four major themes. ‘Regulating normal eating behaviours’, explores the strict eating practices these women engaged in outside of the premenstrual phase. This contrasts with the second theme, ‘Feeding premenstrual cravings and hunger’ which examines constructions of premenstrual cravings and increased hunger. The resulting guilt and positioning of the self as immoral in engaging in ‘bad’ eating behaviours is also discussed in this theme. The third theme, ‘Legitimising a break from feminine body management’ discusses how premenstrual changes and discursive constructions of the premenstrual phase legitimise eating unhealthy food as a form of self-care and comfort. Lastly, ‘Being premenstrual disrupts body sculpting’, explores how premenstrual changes act to inhibit exercise, or as a stimulus for increased exercise associated with heightened negative feelings about the premenstrual body.

### “I eat for fuel”: regulating normal eating behaviours

Outside of the premenstrual phase the majority of women interviewed described their normal eating practices as strictly regulated, conceptualising their bodies and appetites as machine-like. This included consuming “healthy food”, including “fruit”, “salad” and “vegetables”, constructed as ideal eating behaviour. Meal planning and food preparation were also essential to ensure healthy eating and considered to be important in managing the body. Caitlin described pre-preparing meals for weight management and optimal functioning of her body, “So, to me, it’s very important to have my structured meals, make sure I’m getting the right nutrients, the right food, so that my body can function.” Outside of the premenstrual phase she will “eat for fuel. I eat because it’s something that I need to do to get through the day and power myself so that I can actually achieve things and do what I need to do.” Similarly, Rebecca reported being “strict” and “organised” with her food to ensure she has a “certain amount of meals a day”. In these accounts, the woman’s body is positioned as a machine, in need of operation and maintenance with the ‘right’ food to obtain optimal ‘functionality’. This is evidence of biomedical discourses that position food as a means to power the body, rather than as a source of pleasure [49].

A minority of women interviewed also discussed food tracking, as a ‘healthy’ way to monitor calorie, carbohydrate, fat and sugar intake when they were not in the premenstrual phase. Shannon said, “I eat a relatively low carb diet and smaller portion sizes, they’re normally lighter meals, they’re not heavy, they’re not carb heavy.” Tracking food and counting calories are common practices used in the regulation of feminine bodies, discursively positioned as enacting proper femininity [7]. However, these practices can be problematic, as severely restrictive diets constitute disordered eating, associated with the development of an eating disorder [7].

### “I would want to eat pizzas every day”: feeding premenstrual cravings and hunger

Strict regulation of food and a mechanistic conceptualisation of the body was interrupted during the premenstrual phase of the cycle, associated with the majority of participants reporting accounts of increased hunger and cravings for food. For example, Rebecca described that premenstrually, she will “crave food all night and that’s not normally me”. The women discussed craving food they usually denied themselves, including “junk food” and “unhealthy food” such as “pizza”, “cakes”, “chocolate” and “pasta”, contrasted to the “healthy food” that they consumed outside of the premenstrual phase. For example, Ashley described, “During the general period, I usually eat healthy food. I don’t crave for unhealthy food. I mean, I do crave, but it’s not as much. But during premenstrual period, I would want to eat cakes every day or I would want to eat pizzas every day. So, that’s very unusual of me because I’m a person who eats healthy, vegetables five days a week.” The majority of the interview participants and a minority of survey participants positioned their healthy selves outside of the premenstrual phase as their “real” selves and described premenstrual cravings as an interruption of this self and their otherwise controlled eating behaviours. Caitlin positioned herself usual self as “not a chocolate person” describing “if I do crave something sweet, it’s usually something I can resolve with a piece or fruit, something that isn’t as full of unhealthy ingredient”, however premenstrually she can “eat every bit of chocolate in sight.” Similarly, Maria described “I eat quite well. I don’t really have a lot of bad food”, and suggested that she only craves unhealthy food premenstrually, saying “craving is definitely a big factor in why I would eat bad food.” The women in this study may have positioned premenstrual cravings as outside of their true, healthy selves as eating vegetables “five days a week”, choosing “fruit” and avoiding “bad food” is more in line with an acceptable feminine diet, in contrast to “pizza”, “cakes” and “chocolate”, deemed less acceptable or feminine [50]. By positioning premenstrual food cravings as outside of the true self, women maintain their status as healthy and feminine.

Battling food temptation was normalised as a behaviour the women engaged in across the menstrual cycle, described as more difficult premenstrually due to the increased strength of food cravings and hunger, and a weakening of expectations to deny “unhealthy food”. Maria said she was able to combat cravings outside of the premenstrual phase with “fruit or something decent at least because I feel like I don’t normally crave things as much.” However, during the premenstrual phase, she described being more “susceptible to giving in, I think that generally I have more will power and I can say ‘No, I don’t wanna eat that.’ But I feel when I’m premenstrual, I give in to temptation.”

This is in line with previous findings suggesting that women who experience premenstrual cravings are more likely to engage in dietary restraint during the rest of the month [17]. Dietary restraint by avoiding specific foods is indicative of disordered eating [51] and is associated with increased cravings for such foods [52]. This is suggested to have an intrapsychic and physiological basis, as self-regulatory strength and willpower uses energy which becomes depleted following multiple attempts of self-control [53, 54]. Therefore, experiencing heightened cravings for food, along with other physical and emotional premenstrual changes may deplete women’s self-control, and give permission to indulge in “unhealthy” foods that are normally forbidden.

**Fig. 1:**
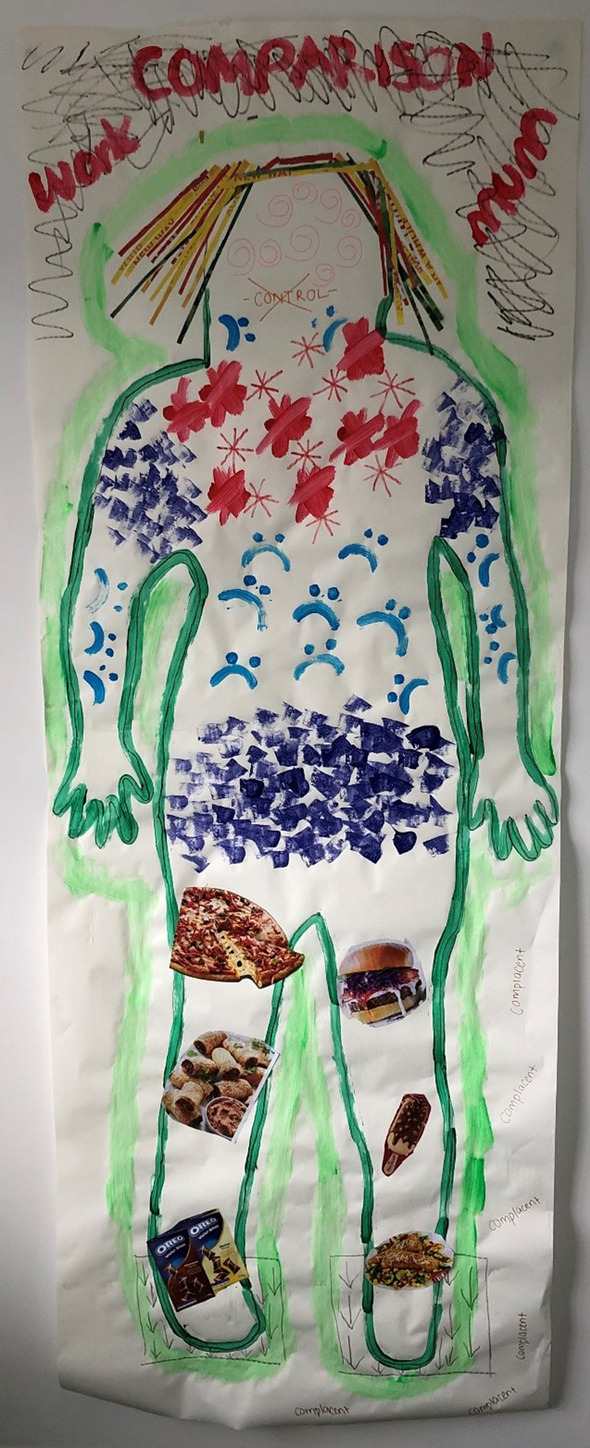
Maria’s body map

Discursive constructions of food also played a role in this process, in that the “junk” food these women craved premenstrually was positioned as “bad food”, in comparison to the “good food” that they ate outside of the premenstrual phase. Maria demonstrated this on her body map, placing images of pizza, a burger, pastries, ice cream and Oreos on her legs, which she positioned as “bad food” (see Fig. 1.). She described, “The food shapes on my legs represent the *bad food* that I crave when I do have PMS. Like the way that all the bad food continues to weigh me down just like the squares with the arrows on my feet.”

In this way, the women attached meaning to their eating behaviours, drawing on binary discursive positioning of food as good (healthy) and bad (unhealthy) [55], which implicitly positions the self as bad for indulging in unhealthy eating. As Lisa said when describing eating chocolate, “I shouldn’t have done that.” Conceptualising food and the self this way has also been found in women with bulimia nervosa [49], indicating disordered eating attitudes in women in the present study.

The majority of survey and interview participants also discussed increased hunger and craving larger portions of food premenstrually. For example, Kristy described feeling “starving”, saying, “I get really hungry and I feel like my portion that I would normally have, I still take lunch for work but I always need something more and I’m always craving something else. I’m eating more than I should and I know that I shouldn’t eat so much, but I do it anyway ‘cause I’m just starving.” For Kristy, eating “so much” included “a big Easter egg”, “leftover pasta” and “chocolate milk”, which she positioned as “out of control”. Kristy’s hunger is suggestive that she is not eating enough to feel sustained, particularly premenstrually, which is often the case with young women who engage in restrictive eating [56]. This suggests a disconnect between expectations of what eating habits should be, and the body’s fluctuating needs across the menstrual cycle.

#### Guilt and immorality associated with the insatiable appetite

Dichotomising food as good or bad had moral implications for these women’s eating behaviours, determining practices that they “should” and “shouldn’t” engage in. In conflating food with social meaning, resistance of food is associated with moral superiority [57], whereas consuming food considered to be ‘bad’ has been suggested to elicit feelings of guilt, particularly in women [58]. Women in the present study reported feelings of guilt in consuming “bad” food they craved premenstrually, as Kristy described “eating a little bit more” during the premenstrual phase is “allowing [her]self to fall into this bad behaviour.” The moral status of the premenstrual self is lowered when the person eats the food that their body may be desiring at the time, if these foods fall into the category of “bad”. This is in line with previous literature that found women who report premenstrual distress self-position as ‘bad’ and outside of ideals of femininity following premenstrual expression of anger or irritation [59]. This discursive construction of the failing feminine self extends to how women position their eating behaviours during the premenstrual phase.

In attaching morality to eating behaviours, some women discussed being undeserving of desirable but “bad” food. The attachment of morality to food is perpetuated by the media within Western culture [58], which for Shannon influenced her feelings of guilt, “I think there’s a lot of influence through the media that’s like, “Oh, you can’t eat any bad food really. You’ve got to eat really well. There’s no treats.” So, that would make me feel very guilty because it’s like—because I was overweight, I didn’t deserve to have something nice or what I was craving, because I should be trying to lose weight, if that makes sense.” In describing herself as overweight, Shannon positioned herself as undeserving of fulfilling her premenstrual cravings, constructing pleasurable food as a “treat” or reward, similarly found in women’s embodied experiences with bulimia [57]. This may reflect an internalisation of fat shaming discourses asserting that overweight people, and particularly overweight women, must constantly strive to make their bodies smaller and therefore ‘healthy’ and acceptable [3]. Fatness itself is positioned as immoral in being associated with poor health, and therefore overweight people are positioned as undisciplined and bad in not following the morally ‘correct’ way to manage their bodies [60]. These discourses provide the context within which women during the premenstrual phase are expected to refrain from enjoying pleasurable food and satisfying their hunger, in order to avoid being positioned as fat, and the distress associated with giving in to the desire to eat.

#### Self-hatred and disgust in not controlling premenstrual cravings

Feeling unable to control and manage cravings during the premenstrual phase was associated with self-hatred and self-disgust. For example, Lisa said resisting her cravings led to her becoming “obsessive” and satisfying them caused her to feel “disgusted” in lacking in self-control, “It might start with ‘I feel like chocolate bar’ and I will hold off on that but then after a little while it almost becomes obsessive. All I can think about is chocolate … then I will go get a chocolate bar but it’s not enough, so then I’ll get a bigger one and I don’t feel okay until I’ve eaten the whole thing but then when I do, I feel disgusted with my lack of self-control.” Caitlin similarly reported “hating” feeling unable to stop herself from eating “a whole bag of mini snickers” premenstrually, describing seeing her body as larger the next day, “I look in the mirror and go, Jesus! You can see where you’ve eaten that big bag yesterday.” Disgust and self-hatred in losing control over one’s eating has previously been reported by women with disordered eating, such as bulimia nervosa [57]. The findings of self-disgust in the present study supports suggestions of an association between premenstrual disorders and eating disorders, with premenstrual distress possibly leading to an exacerbation of bulimia nervosa symptoms [61]. Distress in feeling unable to control one’s eating behaviours due to premenstrual cravings is also associated with body dissatisfaction [5]. For example, a survey participant said, “I’m fat and hate my body” and another shared, “I am more hateful and negative towards my body as I tend to eat more.” This parallels accounts of women reporting bulimia nervosa, wherein binge eating episodes were associated with feeling out of control and body hatred [62, 63]. Exploring experiences with hunger, eating and the discursive positioning of both food and the body may therefore deepen our understanding of both disordered eating and premenstrual distress, and the potential overlap between these syndromes.

**Fig. 2:**
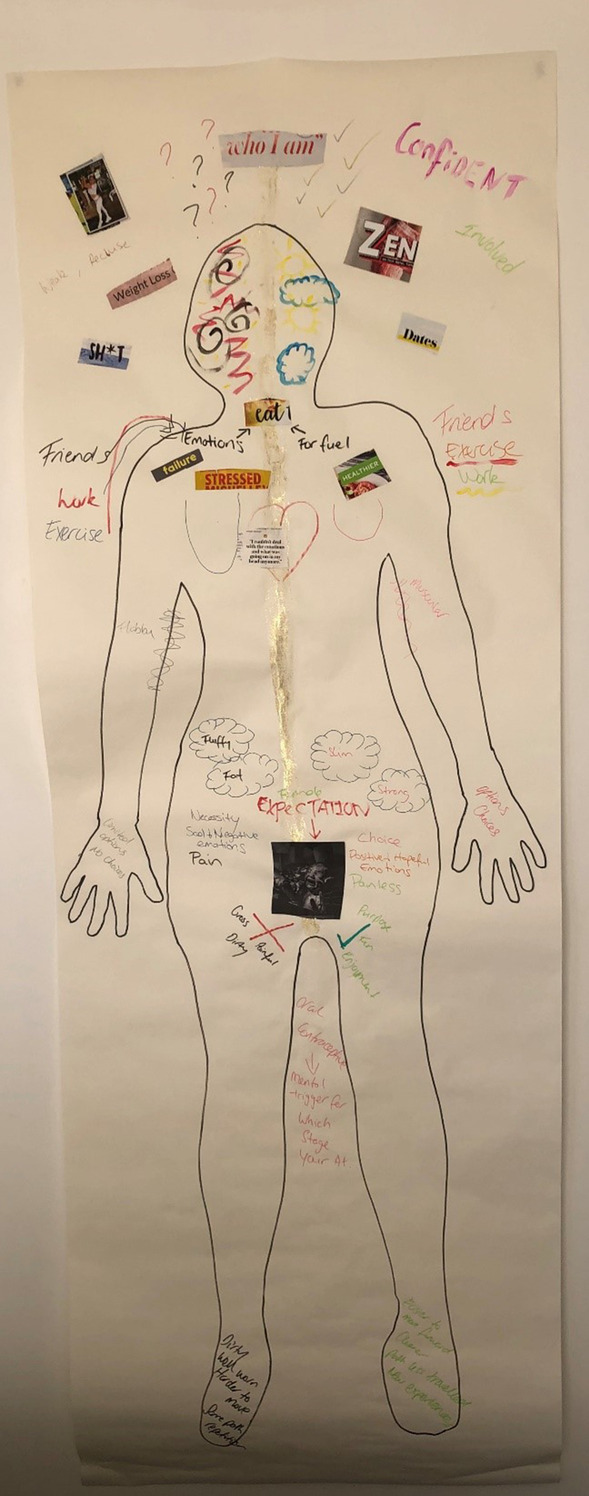
Caitlin’s body map

For the majority of interview participants and a minority of the survey participants, feeling unable to control their eating behaviours was associated with negative emotions towards the self in feeling they were pushing themselves further from obtaining a thin body. Caitlin represented this on her body map in placing an image of a thin female next to her head with the words “weight loss” on the left, premenstrual side of her map (see Fig. 2). She described:

I gotta stop eating ‘cause I’m gonna get fat and it’s just ridiculous to be eating it. I get fairly negative on myself about the way I’m eating compared to how I’d eat the rest of the time because normally it’s so structured. When I’m premenstrual, I’ll grab a bag of liquorice and eat the whole thing. I don’t need it. I guess it goes back to that image of the that thin female. It pulls me further away from that.

Positioning cravings as “ridiculous” suggests that for these women, food is positioned as a tool to be utilised in carrying out the body project of obtaining an idealised thin body. This is reflective of Westernised positioning of the thin body as a symbol of one’s success in conquering bodily desire, and therefore the embodiment of self-control, while the fat body is discursively produced as uncontrolled and gluttonous [64]. Going against these body-management practices may therefore increase these women’s dissatisfaction with their bodies in perceiving themselves as moving away from their ideal body.

### “Because you’re premenstrual, you’re allowed to eat those things”: legitimising a break from feminine body management

This theme discusses how premenstrual changes as well as biomedical discourses and discursive constructions surrounding the premenstrual phase legitimise eating unhealthy food as a form of self-care, comfort and as a means to take a break from strict regimes of body management. For a minority of women, this facilitated reduced self-criticism in eating behaviours, whilst most others experienced feelings of guilt as a result.

#### Situating premenstrual cravings within a biomedical discourse

Some women legitimised “overeating”, eating “junk food”, or being “less strict” with their eating during the premenstrual phase through the adoption of a biomedical discourse that constructs premenstrual changes as being the result of a fluctuation in hormones [16]. These women therefore had a legitimate “reason” and “explanation” for eating more food, or “bad” food, attributing blame for deviation from their usual management of their eating to the premenstrual body, rather than to a failure of the self. For example, Shannon reported “I say it could be my hormones and that’s the reason, it’s okay. I find it helpful to know there’s a reason for it. It lets me attribute blame to something else. It’s not going just on me.” Similarly, Rebecca described, “Usually, I’ll just allow myself to snack all night on whatever. But it doesn’t really change my perception towards myself because I know what I’m doing … I know that it’s not a permanent fault with myself, it’s just that I’m in this hormonal stage … I know that it might be because of my period and not because I’m like a fatty or whatever.” By situating premenstrual embodiment within a biomedical discourse, Rebecca was able to resist the subject position of the “fatty”, an undisciplined woman who does not control and suppress her appetite [7]. Previous premenstrual research suggests women draw upon medicalised regimes of knowledge surrounding premenstrual change in order to explain unfeminine emotions and behaviours [16]. The present findings suggest these women are taking up this subject position to explain deviations from idealised feminine eating practices.

For the minority of interview participants, this biomedical conceptualisation of premenstrual eating behaviours meant it was socially acceptable for women to relax their usual strict eating practices, as this was something that “everyone does”. For example, Whitney described the “societal views” surrounding the menstrual cycle, “I guess, societal views about your period. It’s almost like because you’re premenstrual, you’re allowed to eat those things almost, like it’s the one time of the month that you can … I see it all over social media or your friends talking about—“I’m PMS-ing, so I’m gonna eat this thing that’s really bad.” So I almost feel it’s okay to.” This suggests that the legitimisation of women reducing their management of their eating is influenced by how the premenstrual phase and eating behaviour is positioned within a wider cultural context. These women are drawing upon sociocultural and biomedical discourses that position the reproductive body as out of control and beset by appetites [6], to legitimise more flexible eating patterns and reduce guilt around taking a break from denying their appetites.

#### Feeding cravings under a discourse of self-care

**Fig. 3:**
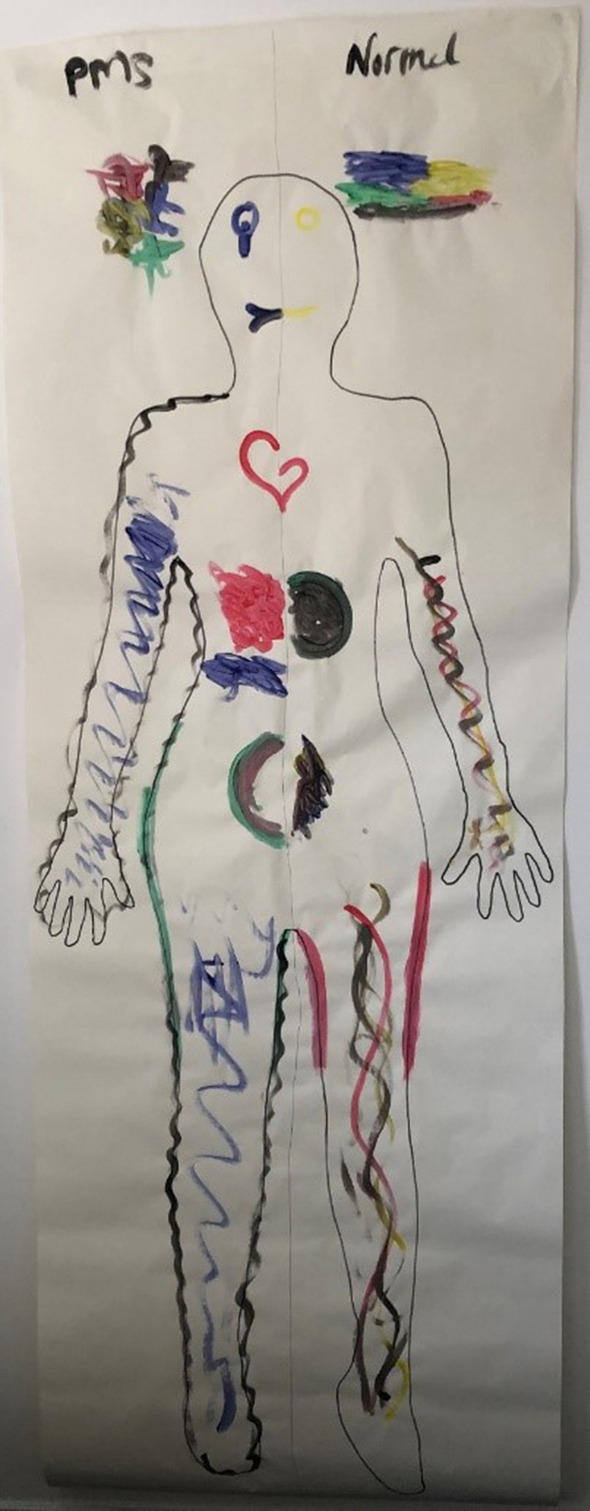
Laura’s body map

For a minority of survey and interview participants, feeling “allowed” to reduce their adherence to strict eating practices premenstrually facilitated engagement in self-care, through responding to their appetite for food and reduced self-criticism associated with eating patterns. Laura represented this on her body map, outlining her stomach in blue on the side representing the premenstrual phase, to signify “increased acceptance” of her hunger during this time and listening to what food her body needs, rather than trying to control her hunger as she does outside of the premenstrual phase (see Fig. 3). She shared:

The blue, I put it there just as my acceptance of the hunger a little bit more. I try to listen to my body more and if it wants more carbs, I give it more carbs, if it wants more fats, I give it more fats, whereas on the normal side I tend to control the hunger a lot more. I don’t really listen to my body that much in my normal life and kind of with that green thing around it as a – I control that part of me a little bit more.

Previous research has reported that discursive constructions of mood change as ‘PMS’ served to legitimise women’s engagement in self-care and taking time out from daily responsibilities [65]. Western discourses of femininity asserting women must put the needs of others before themselves are suggested to lead to a reduced ability in women to monitor their own needs [53]. Women’s constant denial of their appetite for food outside of the premenstrual phase may contribute this reduced ability. In contrast, self-compassion has been associated with lower negative eating attitudes and body shame [66], suggesting practicing acceptance and compassion for premenstrual hunger under a discourse of self-care may reduce distress associated with increased food intake during this time.

For other women, engagement in self-care through reduced regulation of food was legitimised through feeling “uncomfortable” due to negative physical premenstrual changes, positioned as giving them “an excuse” to eat “bad foods”. A survey participant described, “I feel very uncomfortable in my body when I am premenstrual, but at the same time I care less about eating bad foods and being lazy because it is the time when I feel I am allowed to/*it’s my right* to be because I’m uncomfortable. There is a certain kind of *liberation* in that which is conflicting with the sense of being uncomfortable.” Positioning feeling ‘allowed’ to eat bad foods as liberating suggests women’s self-surveillance and management of their eating through constant dieting is exhausting [60]. It also reflects the strength of pressures placed on women to consistently engage in these practices, as despite finding them to be oppressive, women were unable to resist these pressures [3], and only took a break premenstrually. This may be akin to the premenstrual ruptures in self-silencing [67], in which underlying anger associated with enacting feminine ideals is expressed premenstrually [68]. In the context of body management in the present study, women are demonstrating resistance of restrictive management of their appetites.

In conjunction with engaging in self-care in experiencing negative physical changes, a minority of women discussed eating more food and particularly sweet food as a means of coping with negative emotional and psychological premenstrual changes. The embodied experience of eating food was described as providing “comfort” in feeling “irritable”, “stressed” and “grumpy” premenstrually. This is in line with previous research finding that emotional eating increases during the premenstrual phase [69] particularly for sweet and high-fat foods [70]. Ashley allowed herself to indulge cravings for chocolate during the premenstrual phase to manage feelings of sadness, “If I feel sad, then I crave for chocolates. I crave for all the junk food, because again, I feel like *that would make me happy* … so I reach out for everything that I could lay my hands on.” Research has found that restrained eaters are more likely to increase food intake in response to negative emotions, as restraint may become difficult to maintain during psychological distress [71]. Engagement in restrictive eating behaviours outside of the premenstrual phase may have contributed to increased emotional eating associated with negative premenstrual emotions for these women, including negative feelings about the premenstrual body.

Sweet foods such as chocolate and ice cream are typically constructed as comfort foods eaten by women. This is perpetuated through Western media, with representations of women comforting themselves with ice cream being a common image [57]. These gendered discourses were evident in a minority of interview participant accounts as Maria described that “eating [her] feelings” was associated with a feminine stereotype as she said, “it’s like when girls are upset and the stereotype is that they start eating ice cream and they just really eat their feelings.” In drawing upon these discourses, premenstrual comfort eating may also be bound up with constructions of femininity that suggest women are more emotional and irrational than men [57, 64]. These constructions are particularly prominent during the premenstrual phase [59], which may contribute to women in this study positioning themselves within discursive constructions of women as overly emotional and as emotional eaters.

**Fig. 4:**
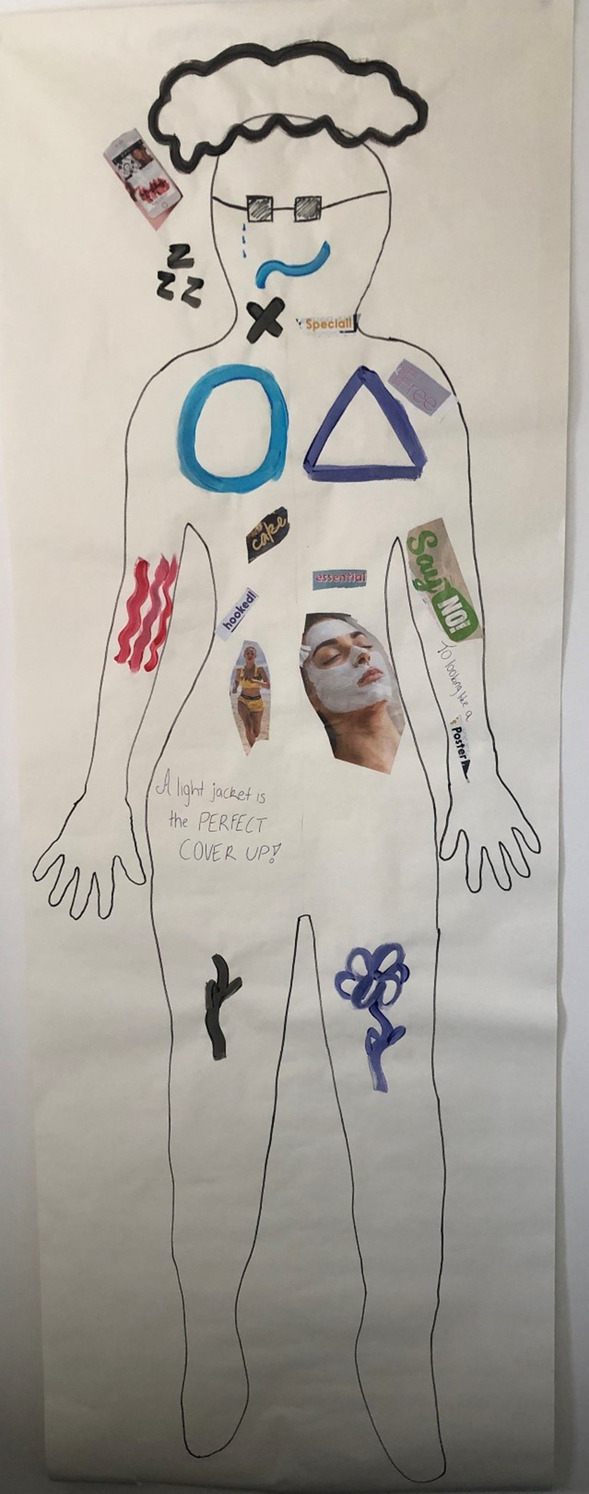
Olivia’s body map

Although the premenstrual phase appeared to legitimise women’s engagement in self-care through fulfilment of their appetites for physical and emotional comfort, a minority of interview participants were unable to completely resist Western cultural discourses that demonise this behaviour, resulting in further emotional distress. These women reported that their improved mood was short-lived, followed by feelings of “regret”, “disappointment” and feeling “worse”. A small number of women described these experiences as a “cycle”, in which eating made them feel better momentarily, followed by an increase in negative feelings in fearing weight gain, which in turn increased their desire to eat comfort food to make themselves feel better. Olivia illustrated this on her body map, painting a blue circle on her chest representing the premenstrual phase, using blue to signify feelings of “self-sadness” and “self-doubt” (see Fig. 4.). She described, “I tend to, when I’m not premenstrual, not eat a lot of chocolate versus when I am premenstrual because I’m like, it tastes so yummy and it makes me feel good, so let’s keep eating this chocolate. But then I think of the self-doubt and self-sadness and then that’s why I put it with the circle of feeling that way.”

Negative cycles created by diets and restrictive eating has been previously documented in research as resulting in harmful cycles of dieting then bingeing, weight loss and regain and feelings of failure [7], with positive impacts of sweet food on emotional state being short lived [72]. Within the context of the premenstrual phase, these short-lived effects may be associated with cultural meanings and implications that construct consumption of sweet and fatty food as problematic in women’s management of their appetites and bodies [56].

### “I should give my body time to rest”: being premenstrual disrupts body sculpting

#### A lack of motivation to sculpt the premenstrual body

**Fig. 5:**
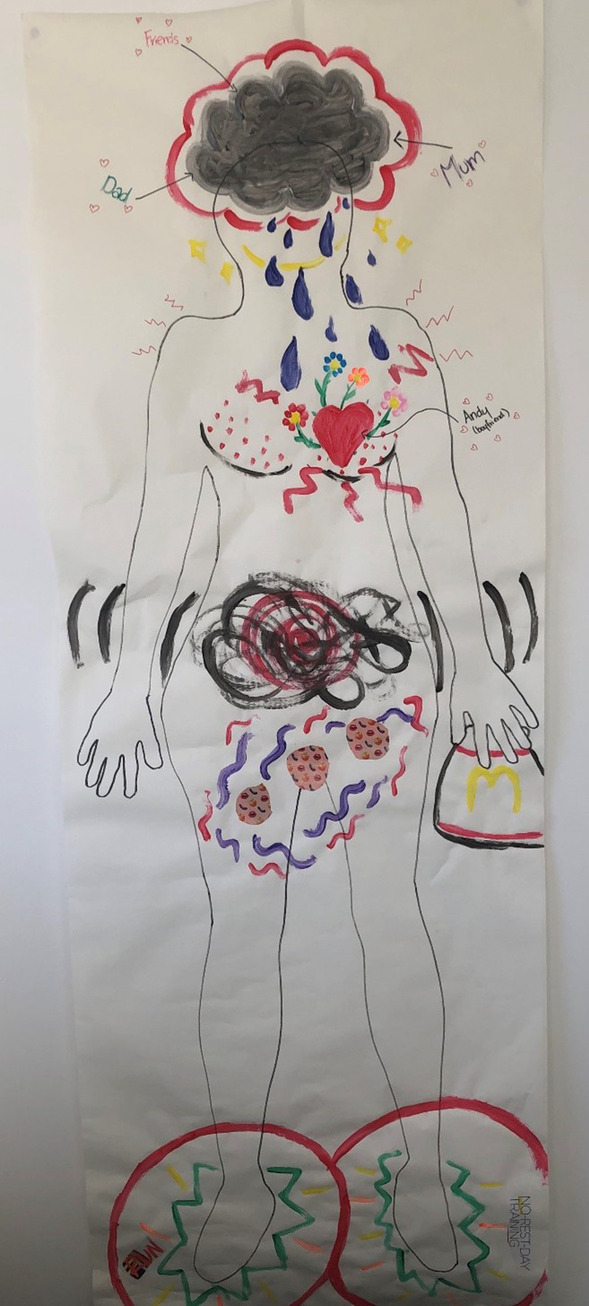
Sarah’s body map

The majority of survey and interview participants reported having strict exercise regimes outside of the premenstrual phase, describing engaging in “high intensity”, “cardio” and “weight-based” workouts most days, or every day, each week. This was associated with positioning themselves and their bodies outside of the premenstrual phase as “strong”, “fit”, “active”, “capable”, “slim” and “healthy”, terms commonly associated with the Western fit body ideal [73]. However, during the premenstrual phase, most of these women described reducing their exercise due to a “loss of motivation”, associated with a combination of negative physical and emotional premenstrual changes such as feeling “depressed” and “upset”, and experiencing “pain”, “fatigue” and being “uncomfortable”. Difficulty in maintaining strict exercise routines when managing premenstrual changes was illustrated by Sarah on her body map, painting red circles over her feet, representing feeling inhibited from continuing her “no rest day training” (see Fig. 5). She described:

Down to my feet, so the little green squiggles and the lines are showing activity, I’ve got little words there, “run” and “no rest day training”. So generally, I like being really active, I’m at the gym five or six days a week. But the red circles there are the premenstrual feelings stopping me from being active I feel like. Like I don’t have the motivation, I just get grumpy and tired and unmotivated.

This demonstrates that engaging in intensive exercise each day may be a difficult task, requiring a significant amount of energy and work [74], becoming increasingly difficult when managing premenstrual changes.

In describing physical and emotional premenstrual changes as disrupting motivation to exercise, a minority of survey and interview participants positioned their premenstrual bodies as “weak”, “less capable”, and “slow” associated with increased negative feelings about the body. Michelle associated exercise with her body feeling “capable” and “comfortable” as opposed to how she feels premenstrually, “I think when you are active, you feel capable again and you feel better about your body, feel more comfortable. You feel healthier. You feel like your body is performing the way it’s *supposed to* and when you’re not, it’s the opposite.” Constructions of the premenstrual body as faulty, weak, incapable and slow are comparable to the ways in which the fat, unhealthy body is discursively positioned within Western culture [60]. Positioning unfailing motivation to exercise as normative for women is also evidence of internalisation of discourses asserting that women must consistently engage in body sculpting behaviour [8]. Premenstrual pain and fatigue may disrupt women’s ability to assume the subject position of an individual who manages their body through exercise, consequently inhibiting women’s ability to adhere to constructions of idealised health and femininity.

#### Feelings of guilt and the lazy premenstrual body

An implication of women feeling unmotivated and reducing exercise during the premenstrual phase was experiences of guilt, and distress in not striving for a thin body. Feelings of guilt in missing exercise have been documented within previous research [75], found to be more prevalent in women who exercise for appearance and weight-control rather than health reasons [76] and is associated with body dissatisfaction and disordered eating [77]. Exercising for appearance-related reasons was evident in Sarah’s account, as she discussed feeling guilty in not striving to change her body and consequently achieve happiness during the premenstrual phase, “At the time, I’ll be telling myself, “Oh, I don’t need to work out. I’ll just have a rest.” But then the guilt kind of settles in and I start thinking, “Well, if you want to change yourself and you wanna be happy and you want to lose weight, you need to be working out.” Women declared than an ideal body is attainable if they engage in strict exercise and body discipline, reflecting popular discourse surrounding women’s body regulation [78]. This ultimately sets women up for feelings of failure, as this body is difficult and impossible for a lot of women to achieve [8]. In experiencing material premenstrual changes such as bloating, pain and fatigue, women may perceive obtaining the body ideal through exercise as increasingly difficult, contributing to premenstrual distress.

Feeling “disappointed” and positioning the self as “lazy” and useless” in missing exercise was also evident in a minority of survey and interview participant accounts. Whitney discussed feeling “useless” and “disappointed”, blaming herself for not being able to push through premenstrual fatigue and continue her usual exercise, “I become too fatigued and unmotivated to continue my normal workouts so that causes distress and guilt. I feel useless probably. I just am disappointed in myself too because I think that—just knowing that I can do it, but I’m not doing it—it’s frustrating.” Many women blame themselves for not exercising enough, rather than criticising the substantial pressure placed on women to sculpt their bodies into a culturally acceptable shape [8]. Constructing missing of exercise as ‘laziness’ means that women were not legitimated in resting or taking a break from body sculpting in response to premenstrual changes in pain, fatigue or emotions. Laziness is discursively positioned as a lack of commitment to changing and controlling the body [3]. Internalisation of these discourses had negative intrapsychic consequences for how women positioned resting their bodies during the premenstrual phase and feelings about the body and the self.

#### Increased body sculpting due to fear of fatness

In contrast to reducing exercise during the premenstrual phase, a minority of interview and survey participants reported increasing their exercise, due to perceived premenstrual fatness and increased negative feelings about the body. Participants reported “push[ing] through” in prioritising and increasing exercise, despite experiencing embodied premenstrual changes that made exercising more difficult and physically and emotionally taxing. Olivia illustrated this on her body map, placing an image of a “fitness model” along with the word “hooked!” on her stomach on the left, premenstrual side of her map. This was to demonstrate her feeling hooked on exercising to reach thin bodily ideals perpetuated by the media (see Fig. 5). She discussed a battle between her mind wanting to increase exercise to achieve this ideal, and her body wanting to rest due to premenstrual fatigue, “I feel so sluggish and slow and heavy, so then I feel like, “Why am I even here?” I’m hooked on those thoughts, on images of looking like a fitness model when obviously my body is just also trying to say to me, “You need to rest and slow down, but I want you to look like this”. Feeling pressure from the media to be thin has been found to be associated with compulsive exercise in girls [79]. Exercising for appearance and weight management has also been found to be negatively associated with fitness and health management [80]. This is evident in these accounts, in these women’s denial of their body’s need to rest in experiencing premenstrual changes and instead pushing the body in favour of attempting to manage their weight.

For these women, pushing the body to exercise during the premenstrual phase was associated with making them feel “worse”, “guilty” and increasing premenstrual distress, discussed as ineffective in reducing body dissatisfaction. For example, Whitney shared, “I push myself even harder even though I’m feeling more tired. And in the end, I feel ten times worse because I’m so tired but still dissatisfied. I should give my body time to rest.” Fitness and weight loss messages within the media promise women that working towards and achieving a thin body will make them feel confident and empower them in taking control of their bodies [78]. However, this instead encourages women to focus on their presumed flaws and work on them [78]. Increased dissatisfaction with the premenstrual body may further exacerbate this negative focus and in turn increase motivation to ‘fix’ perceived bodily flaws and push the body beyond its physical limits, in turn making women feel worse.

## Conclusions

This study examined how women who report premenstrual body dissatisfaction construct and experience changes to eating and exercise behaviours during the premenstrual phase, in order to provide greater understanding of premenstrual body dissatisfaction and distress. The findings of this study suggest that cultural ideals of feminine body management, which position cravings, hunger and the need to rest the body as unfeminine, play a role in premenstrual distress and body dissatisfaction. These cultural ideals had implications for how these women negotiated changes to their usual mechanistic body management behaviours in the premenstrual phase of the cycle. For women who experience premenstrual body dissatisfaction, the premenstrual phase may be a time that they allow themselves to take a break from engaging in restrictive eating and intensive exercise in pursuit of the thin, toned body ideal. This facilitated engagement in self-care through listening to and caring for the body’s needs by allowing for increased food intake and resting. However, for others, this was associated with negative psychological and physical consequences, manifested in guilt, shame, self-disgust, and pushing the body physically through increased exercise.

This suggests that for women who feel negatively about their premenstrual bodies, premenstrual embodiment is complex and multi-faceted. Fluctuations in the ways in which these women manage their eating and exercise needs to be considered in understanding premenstrual body dissatisfaction and distress. These findings also have implications for women’s disordered eating and exercise behaviours, in suggesting that one’s management and negotiation of these behaviours may be influenced by changes across the menstrual cycle. It also suggests that premenstrual body dissatisfaction and distress may play a role in women’s disordered eating and exercise behaviours and should be acknowledged as a possible contributor within clinical settings. Insights gained from these research findings also suggest that women may benefit from receiving information and support regarding premenstrual changes to eating and exercise behaviours and premenstrual body dissatisfaction, such as within school and community education settings. Future in-depth research is needed to explore the role that discursive constructions of feminine body management in the context of premenstrual changes have on women’s disordered eating and exercise behaviours. Further research should also examine the implications of premenstrual body dissatisfaction, premenstrual distress and fluctuations in body management behaviours in women with eating disorder diagnoses. Examining women’s eating and exercise behaviours as well as their feelings about the body at multiple times across the menstrual cycle, rather than retrospectively, may also provide greater in-depth insight into these changes.

Strengths of this study include the use of a community sample and arts-based qualitative methodology, facilitating a greater depth of understanding of women’s experiences with their premenstrual bodies, in the context of eating and exercise behaviours. Limitations of this study were that participants responded to an advertisement asking about negative feelings about the premenstrual body, and thus we excluded women who do not experience negative feelings about the premenstrual body. Another limitation is that participants were predominantly young, cisgender, heterosexual, Caucasian women and further research is needed to examine premenstrual embodiment on older women, non-heterosexual, and women from other ethnic groups. Although participants completed a standardised measure of disordered eating attitudes, they were not screened for past or present eating disorders which may have provided insight into the relationship between premenstrual body dissatisfaction and eating disorders.

These findings demonstrate the harsh cultural pressures placed on women to control and discipline their bodies through restrictive eating behaviours and rigorous exercise, a process which is further complicated by premenstrual changes and distress. How women negotiate their premenstrual bodies in relation to discourse around acceptable femininity therefore has material implications for women’s premenstrual body dissatisfaction and distress.

## Data Availability

Data sharing is not applicable to this article due to protection of participant privacy in that participants consented for their data to be used by the researchers and similar studies conducted at Western Sydney University only.

## Abbreviations

PMS: Premenstrual syndrome
PMDD: Premenstrual dysphoric disorder
PSST: Premenstrual Symptom Screening Tool
EAT-8: Eating Attitudes Test 8
OBCBSS: The Objectified Body Consciousness Body Shame Subscale
